# Stakeholder involvement in systematic reviews: a protocol for a systematic review of methods, outcomes and effects

**DOI:** 10.1186/s40900-017-0060-4

**Published:** 2017-04-21

**Authors:** Alex Pollock, Pauline Campbell, Caroline Struthers, Anneliese Synnot, Jack Nunn, Sophie Hill, Heather Goodare, Chris Watts, Richard Morley

**Affiliations:** 1 Nursing Midwifery and Allied Health Professions (NMAHP) Research Unit, Glasgow Caledonian University, Cowcaddens Road, Glasgow, G4 0BA Scotland; 2 Education and Training Manager, EQUATOR Network, Centre for Statistics in Medicine, NDORMS, University of Oxford, Botnar Research Centre, Windmill Road, Oxford, OX3 7LD UK; 3 Cochrane Consumers and Communication, Centre for Health Communication and Participation, School of Psychology and Public Health, La Trobe University, Kingsbury Drive, Bundoora, VIC 3086 Australia; 4 Cochrane Australia, School of Public Health and Preventive Medicine, Monash University, L1, 549 St Kilda Road, Melbourne, VIC 3004 Australia; 5 Edinburgh, UK; 6 Cochrane Learning and Support Department, Cochrane Central Executive, St Albans House, 57-59 Haymarket, London, SW1Y 4QX UK; 7 Cochrane Consumer Network, St Albans House, 57-59 Haymarket, London, SW1Y 4QX UK

**Keywords:** Systematic review, Evidence synthesis, Involvement, Stakeholder, Patient, Public, Consumer

## Abstract

**Plain English Summary:**

Researchers are expected to actively involve stakeholders (including patients, the public, health professionals, and others) in their research. Although researchers increasingly recognise that this is good practice, there is limited practical guidance about how to involve stakeholders. Systematic reviews are a research method in which international literature is brought together, using carefully designed and rigorous methods to answer a specified question about healthcare. We want to investigate how researchers have involved stakeholders in systematic reviews, and how involvement has potentially affected the quality and impact of reviews. We plan to bring this information together by searching and reviewing the literature for reports of stakeholder involvement in systematic reviews. This paper describes in detail the methods that we plan to use to do this.

After carrying out comprehensive searches for literature, we will:

1. Provide an overview of identified reports, describing key information such as types of stakeholders involved, and how.

2. Pick out reports of involvement which include detailed descriptions of *how* researchers involved people in a systematic review and summarise the methods they used. We will consider who was involved, how people were recruited, and how the involvement was organised and managed.

3. Bring together any reports which have explored the effect, or impact, of involving stakeholders in a systematic review. We will assess the quality of these reports, and summarise their findings.

Once completed, our review will be used to produce training resources aimed at helping researchers to improve ways of involving stakeholders in systematic reviews.

**Abstract:**

**Background**

There is an expectation for stakeholders (including patients, the public, health professionals, and others) to be involved in research. Researchers are increasingly recognising that it is good practice to involve stakeholders in systematic reviews. There is currently a lack of evidence about (A) *how* to do this and (B) the effects, or impact, of such involvement. We aim to create a map of the evidence relating to stakeholder involvement in systematic reviews, and use this evidence to address the two points above.

**Methods**

We will complete a mixed-method synthesis of the evidence, first completing a scoping review to create a broad map of evidence relating to stakeholder involvement in systematic reviews, and secondly completing two contingent syntheses. We will use a stepwise approach to searching; the initial step will include comprehensive searches of electronic databases, including CENTRAL, AMED, Embase, Medline, Cinahl and other databases, supplemented with pre-defined hand-searching and contacting authors. Two reviewers will undertake each review task (i.e., screening, data extraction) using standard systematic review processes.

For the scoping review, we will include any paper, regardless of publication status or study design, which investigates, reports or discusses involvement in a systematic review. Included papers will be summarised within structured tables. Criteria for judging the focus and comprehensiveness of the description of methods of involvement will be applied, informing which papers are included within the two contingent syntheses.

Synthesis A will detail the methods that have been used to involve stakeholders in systematic reviews. Papers from the scoping review that are judged to provide an adequate description of methods or approaches will be included. Details of the methods of involvement will be extracted from included papers using pre-defined headings, presented in tables and described narratively.

Synthesis B will include studies that explore the effect of stakeholder involvement on the quality, relevance or impact of a systematic review, as identified from the scoping review. Study quality will be appraised, data extracted and synthesised within tables.

**Discussion**

This review should help researchers select, improve and evaluate methods of involving stakeholders in systematic reviews. Review findings will contribute to Cochrane training resources.

**Electronic supplementary material:**

The online version of this article (doi:10.1186/s40900-017-0060-4) contains supplementary material, which is available to authorized users.

## Background

The concept of active involvement in research of stakeholders was founded on the principle that the public have a moral right to contribute to decisions about what research is undertaken and in what way [1–3]. We define stakeholders as *any potential knowledge user whose primary job is not directly in research* [1], including people with a healthcare condition, their families, friends and caregivers, health professionals, decision makers and others. It is now widely accepted that active stakeholder involvement is beneficial to the quality, relevance and impact of health research [2, 3], and this has driven national strategies in many countries to ensure involvement in all research activities [4]. There is now an expectation from funding bodies, including government and charities, that researchers will actively involve patients and the public in their research, including systematic reviews [2, 5–8].

Systematic reviews aim to inform and support the delivery of evidence-based practice, by finding and bringing together, in an explicit and transparent way, all the research evidence that addresses a particular topic or healthcare question. Active stakeholder involvement within systematic reviews has been proposed as a way to enhance the actual and perceived usefulness of synthesised research evidence, addressing barriers to the uptake of evidence into practice [9]. While there are a number of examples of active stakeholder involvement in systematic reviews, the approaches to, and extent of, involvement have varied considerably [5, 10, 11]. Cochrane, an international organisation which produces systematic reviews of healthcare evidence, has had patient and public (described by Cochrane as “consumer”) involvement as an explicit principle of the organisation since it began in 1993 [12, 13]. However a recent review of Cochrane consumer activity concluded that while Cochrane consumer contributors comment on protocols, reviews and plain language summaries, there are few examples of active consumer involvement in the conduct of Cochrane reviews [5, 14]. This review focussed on consumer involvement at the organisational level (with an emphasis on Cochrane), rather than activities and roles of individual researchers and how they may involve stakeholders in their reviews. Thus there remains a lack of evidence about the best ways to actively involve stakeholders in systematic reviews [15], and the impact of involvement on research activity and uptake of evidence [2].

The aims of this systematic review are to:

1. find and bring together evidence relating to stakeholder involvement in systematic reviews in order to provide a broad map of the current evidence-base (a scoping review), and,
2. use this evidence to:
  A. Describe the methods or approaches which have been used in relation to stakeholder involvement in systematic reviews
  B. Summarise the evidence relating to the effect of stakeholder involvement on the quality, relevance or impact of systematic reviews

## Methods

### Design

**Fig. 1:**
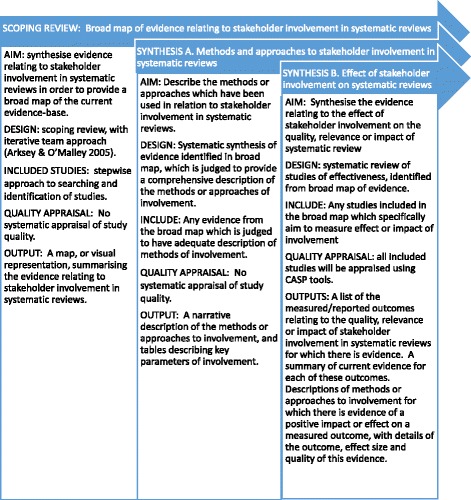
Outline of contingent review design

We plan a mixed method evidence synthesis, using a contingent design to systematically map and assimilate evidence in relation to our research objectives. Systematic maps of research evidence are useful for informing the planning, conduct and interpretation of an evidence synthesis [16]. Contingent designs comprise a cycle of research syntheses, conducted to address clearly defined objectives, and assimilate evidence according to its relevance to a clear objective or question, rather than grouping studies according to whether they have a qualitative or quantitative research design [17]. An outline of the planned contingent design is illustrated in Fig. 1. The design will therefore incorporate a broad map of evidence relating to stakeholder involvement in systematic reviews (a scoping review, [18, 19]), followed by two contingent syntheses each specifically addressing a different research objective.

**Fig. 2:**
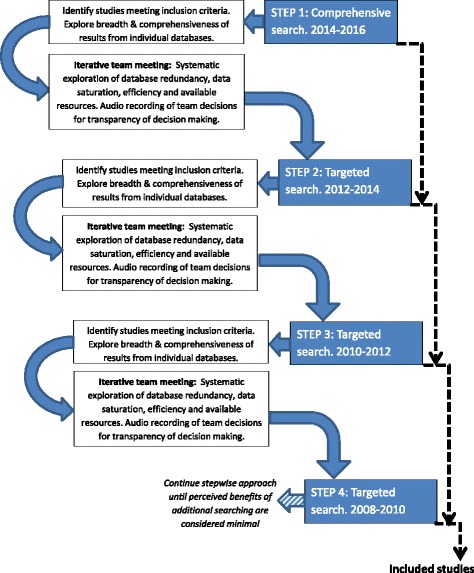
Stepwise approach to searching

For the scoping review, and following the Arksey and O’Malley (2005) framework, we will use an iterative team approach to ensure clarity of purpose and balance between breadth and comprehensiveness of the review [18, 20]. In addition to traditional scoping review methods, in order to facilitate efficient identification of relevant up-to-date literature, we will implement a stepwise approach [21] to the identification of literature for inclusion. This approach involves a series of pre-planned searches, with progression from one stage (or ‘step’) to the next dependent on consideration of the results of the previous step, aimed at enabling efficient identification of the most relevant evidence. Pre-agreed criteria and contingencies will be used to inform discussions and reach consensus on whether to progress to the next step of searching, and to define the parameters of that search, or whether to cease searching. Year of publication has been selected as central to the proposed searching steps as there is evidence of rapid changes in stakeholder involvement in research over time [6], and we anticipate an increase over time in reported stakeholder involvement in systematic reviews. The proposed stepwise approach to searching for the scoping review is illustrated in Fig. 2. We acknowledge that this is a novel approach to searching, and we hope to learn methodological lessons from use of this approach.

### Scoping review methods

#### Search methods: step 1

Step 1 of the stepwise search strategy will involve comprehensive searching of electronic databases [22], from 01/01/2014 - current date 2016, and will be supplemented with searching other sources. Databases will include CENTRAL (CDSR, DARE, HTA, Cochrane Methodology Register), AMED (OVID), DoPHER (EPPI-centre database), Embase (OVID), Medline (OVID), CINAHL (EBSCO) and Joanna Briggs database. A comprehensive search strategy will be used, adapted for each database, using established search filters. An example search strategy is provided in Additional file 1.

Other searching to be included in Step 1 will include hand searching of ProQuest Dissertations & Theses (UK & Ireland), Epistemonikos, PDQ-Evidence and Research Involvement and Engagement journal. We will consider the papers identified in Cochrane Consumer Function Review [14], the reference lists of known reviews relating to involvement e.g., [6, 23], and the reference lists of all included studies. In order to identify unpublished data, expert experience and ‘grey’ literature relating to involvement in systematic reviews, we will contact authors of published papers, contact relevant organisations and promote this review via social media.

#### Search methods: subsequent steps

To inform the team decision as to whether to progress to the next step of searching, and if so the details of the search strategy for the next step, data will be collected on the number of unique references identified for inclusion from each source searched. Redundancy of individual databases will be considered in relation to the number of unique citations judged to meet inclusion criteria. Efficiency will be considered by exploring the proportion of papers included in relation to the number of titles within the search results from each individual database. These data will inform the iterative team decisions for subsequent steps, which will consider expanding the searches of each individual source by a further 2 years. This process will continue in a cyclical fashion, until it is perceived that the optimal breadth and comprehensiveness of evidence has been identified, with consideration of data saturation and available project resources (Fig. 2). To ensure transparency of the iterative team decisions, we will audio record each of the meetings and will report key themes related to all decision making.

#### Criteria for inclusion in the scoping review

Selection criteria for inclusion in the broad map of research evidence will be purposefully wide. We will include any paper, published or unpublished, regardless of study design, including commentaries, letters and expert opinion, which investigates, reports or discusses any aspect of involvement in a systematic review.

##### Types of evidence

We anticipate that we will include (but will not be limited to) the following types of evidence:

- Published systematic reviews which report involvement
- Reports of methods of involvement in an individual systematic review
- Reports of methods of involvement in an organisation which commissions, undertakes or supports systematic reviews
- Studies quantitatively or qualitatively evaluating involvement in individual systematic reviews, or in organisations which commission, undertake or support systematic reviews
- Opinions, commentary and discussion relating to involvement in systematic reviews or organisations that commission, undertake or support systematic reviews

##### Definition of stakeholder

As stated above, we define a stakeholder as *any potential knowledge user whose primary job is not directly in research*. Potential knowledge users include a broad range of people, including those who are actual or potential recipients of health or social care, where this may include patients, carers and family members, or people interested in remaining healthy who are seeking information about a health condition or treatment for personal use [24]; members of organisations that represent people who use services; people with a professional role in health and social care; policy makers and managers. Given the variety of terms used to describe stakeholders (e.g., “consumers”, “patients and the public”), the different types of stakeholders who could be involved, and the importance of distinguishing between the perspectives of the public and the perspectives of people who have a professional role [25], *w*e will document, categorise and report the types of people involved within any evidence included in this review.

##### Definition of systematic review

We will define a systematic review as a research process in which literature relevant to a stated question is identified and brought together (synthesised) using explicit methods [26], including reporting of inclusion/exclusion criteria, search methods and details of included studies. We will include studies which report involvement in systematic reviews regardless of the type of evidence synthesised in the systematic review (i.e., quantitative, qualitative, mixed-methods) and the type of question addressed (e.g., intervention effectiveness, diagnostic test accuracy, patient experiences).

##### Definition of involvement in a systematic review

We will apply a wide definition to stakeholder involvement in a systematic review (or organisation which commissions, undertakes or supports systematic reviews). We will include reports relating to any role or contribution toward the development of a review protocol, completion of any of the stages of a systematic review, or dissemination of the findings of a review.

We will exclude:

- Reports of involvement focused specifically on the generation of research priorities and questions, unless these are explicitly questions for systematic reviews.
- Systematic reviews focused on synthesising evidence relating to stakeholder involvement in primary research.
- Discussion and commentary relating to stakeholder involvement in research more broadly, or in guideline development, unless there is explicit mention of involvement in systematic reviews.

### Methods of selection of studies

One member of the review team (PC) will run the search strategy and exclude any obviously irrelevant titles. Two review authors (PC, AP) will independently review the abstracts of all remaining records, applying selection criteria to identify eligible studies. Full papers will be obtained for all studies considered potentially relevant by at least one reviewer, and will be independently assessed by two reviewers. Any disagreements between reviewers will be resolved through discussion, involving a third reviewer (CS) where necessary.

### Data extraction and mapping the evidence

Data will be extracted into structured tables, categorising each included paper as either:

A. Report specifically focused on the methods of, or effect of, stakeholder involvement in a systematic review(s)
B. A systematic review that reports stakeholder involvement in the review process
C. Other report that describes, discusses or comments on stakeholder involvement in a systematic review(s)
D. Unpublished data or expert experience relating to stakeholder involvement in systematic reviews

One reviewer will extract the following data, and a second reviewer will independently check the data entry:

- Bibliographic information
- Year of publication
- Publication status (published/unpublished)
- Methodological focus/study methodology
- Description of reported method(s) or approach(es) to involvement of members of public
- Details/experience of people involved (patients, carers, professionals, policy makers etc.)

Two reviewers will independently judge the focus and comprehensiveness of each of the included papers, using the criteria below (Table 1) (adapted from [27]). Where there are disagreements between the two reviewers, this will be resolved through discussion with the involvement of a third reviewer where necessary.

**Table 1:** Criteria for judgement of focus and comprehensiveness of reports of involvement in systematic reviews

Comprehensiveness Focus	‘Green’	‘Amber’	‘Red’	Not applicable
Methods or approaches to involvement in systematic reviews	Comprehensive description of one (or more) specific method or approach to the involvement in systematic reviews. Description sufficient to enable replication of methods.	A brief or partial description of one (or more) specific method or approach to the involvement in systematic reviews. Description sufficient to enable partial replication of methods.	Few details provided and/or inadequate description of the method or approach of involvement. Description insufficient to enable any replication of methods.	Paper does not aim to describe a particular method or approach to involvement.
Study of effectiveness	Reports a study which was specifically designed to explore the effect of involvement (on any outcome), including a comprehensive description of study design or methods.	Reports data which relates to measures of the effect of involvement (on any outcome), but inadequate description of study design or methods.	States or discusses outcomes relating to effect of involvement, but without measuring the effect, or without reporting how outcomes were measured.	Paper not focused on studying effectiveness.

These data will be presented within tables, and a visual representation of the volume and comprehensiveness of evidence provided. The tabulated data on the breadth and comprehensiveness of each of the included papers will inform iterative team discussions regarding stepwise expansion of the searches. Following completion of the final step of searching, the complete tabulated dataset will be used to determine which papers are included within the syntheses focused on (A) methods and approaches to involvement, (B) evidence of effect of involvement. The methods specific to each of these two syntheses are described below

### Synthesis A methods

#### Included studies

Studies from the scoping review that are judged as ‘green’ or ‘amber’ for comprehensiveness of description of methods or approaches to involvement (table 1) will be included.

#### Data extraction and synthesis

A narrative description of the methods or approaches to involvement will be extracted. In addition, we will tabulate the following information:

- Stated aim of involvement
- Number of people involved
- Characteristics of people involved
- How people were recruited
- Format of involvement (face-to-face meeting, telephone meeting, written consultation, online survey, other)
- Stage(s) of review at which there was involvement (question, protocol, search & identification of studies, data extraction, quality appraisal & synthesis, data interpretation, dissemination)
- Amount of involvement (number of meetings, number of days involved)
- Formal research methods used (e.g., participatory action research, nominal group technique, Delphi method)
- Any evaluation of the methods which was done
- Ethical approval obtained for involvement?
- Financial compensation (or alternative) for people involved?
- Tools or method of reporting involvement?

The quality of the studies contributing to this synthesis will not be systematically appraised, as this is not contingent with the aim of this synthesis, which is focused on providing a description of methods or approaches of involvement (rather than synthesising or interpreting outcome data).

### Synthesis B methods

#### Included studies

All papers previously identified, as ‘studies of effectiveness’ will be included, regardless of whether they were judged as green, amber or red for comprehensiveness. This approach will be taken for this synthesis as the quality of the study or effectiveness will be assessed, and subsequent decisions will be based on quality of evidence (rather than comprehensiveness of reporting).

We will include any study designs, including experimental and observational studies. We will include studies with or without a control intervention.

#### Appraisal of study quality

Two independent reviewers (AP, PC) will assess and report the quality of all studies of effectiveness using CASP appraisal tools [28], with any disagreements resolved through discussion, involving a third reviewer (CS) if necessary.

#### Data extraction and synthesis

We will extract data into structured tables, including the following:

- Study aim
- Study design
- Focus of systematic review
- Summary of involvement – including numbers, who was involved, at what stages in the review process, and methods used.
- Outcomes assessed, relating to the effect of involvement
- Results (measured effect or impact of involvement)

The different types of outcomes assessed will be synthesised, creating one list of unique methods of assessing the effect of involvement in systematic reviews. These methods will be categorised as measures of review quality, relevance of review, impact of review, or other effect. Any data relating to the psychometric properties, benefits or limitations of these outcomes will be systematically tabulated from the included studies.

For each of the included studies, the measured effect on the identified outcome measures will be documented. This may comprise quantitative or qualitative outcome data. A summary of the judgement of the quality of the study contributing the data will be incorporated into the table. A summary of findings table will be produced, summarising any evidence for effect on the measured outcomes, for different methods or approaches to stakeholder involvement. We will write a description of the reported beneficial effects of involvement, using a similar format to that used by Brett 2014 when describing the impact of patient and public involvement on health and social care research [29].

### Integration of data from syntheses A and B

We will identify studies that were (i) judged to have a ‘green’ or ‘amber’ comprehensive description of the methods or approach (in synthesis A) and (ii) demonstrated evidence of a beneficial effect (in synthesis B). For these studies we will tabulate the key components of the methods or approaches, identifying areas of agreement or dissonance between studies. We will contact the authors of all of these studies and seek any additional material or resources associated with the method or approach.

## Discussion

To ensure that stakeholder involvement is beneficial to the quality, relevance and impact of systematic reviews, it is essential to have clearly described approaches to involvement. This must be supported by evidence relating to the effect or impact associated with involvement, as well as effective strategies for measuring the impact of involvement on the uptake and use of systematic review evidence. This planned systematic review will synthesise evidence relating to stakeholder involvement in systematic reviews. Using a contingent design, an initial scoping review will provide a broad map of evidence relating to involvement, with two more focused syntheses to describe the methods and approaches to involvement that have been used, and to explore the evidence of effectiveness of involvement. These syntheses will identify the current methods that have been used to assess the effect of involvement on systematic reviews, and summarise the evidence of effectiveness for these outcomes.

Use of an innovative stepwise search strategy will ensure the balance of breadth and comprehensiveness of data within a review for which searching and identification of relevant studies is anticipated to be challenging. Audio-recording and documentation of iterative team decisions associated with the stepwise and scoping methods will ensure transparency in the review process. Contacting researchers and stakeholders involved in systematic reviews in which there is evidence of a beneficial effect of involvement will enable a richer description of methods and approaches, providing descriptions which can support future replication and improvement.

There is widespread consensus that high-quality training material, reporting guidelines and examples of best practice are urgently required to support active patient and public involvement and enhance the relevance, usefulness and accessibility of systematic reviews [3, 11, 15, 30, 31]. When we have completed our review, the findings will be used to produce training material and resources, as part of the ACTIVE project (http://training.cochrane.org/ACTIVE), which is being carried out in collaboration with Cochrane Training. The focus of these resources will be the production of clearly described methods of *how* stakeholders may be usefully involved in systematic reviews. We encourage anyone with knowledge, information or experience relating to stakeholder involvement in systematic reviews to contact the ACTIVE project, via the project website or email (ACTIVE@gcu.ac.uk).

## Additional file

Additional file 1: Medline Search strategy (Ovid). (DOCX 23 kb)

## Abbreviations

ACTIVE: Authors and Consumers Together Impacting on eVidencE
AMED: Allied and Complementary Medicine Database
CDSR: Cochrane Database of Systematic Reviews
CENTRAL: Cochrane Central Register of Controlled Trials
CINAHL: Cumulative Index to Nursing and Allied Health Literature
DARE: Database of Abstracts of Reviews of Effectiveness
DoPHER: Database of Promoting Health Effectiveness Reviews
EBSCO: An online platform for accessing research databases
Embase: Excerpta Medica Database
EPPI-centre: Evidence for Policy and Practice Information and Co-ordinating Centre
HTA: Health Technology Assessment
Medline: On-line Medical Library database
OVID: An online platform for accessing research databases
PDQ-Evidence: (“Pretty darn quick”) database of systematic reviews of health systems evidence
