# Supplementary material for: Stakeholder involvement in systematic reviews: a protocol for a systematic review of methods, outcomes and effects
Source: Res Involv Engagem. 2017 Apr 21;3:9. doi: 10.1186/s40900-017-0060-4 (PMC5611627; doi:10.1186/s40900-017-0060-4)
Supplement: Additional file 1: — Medline Search strategy (Ovid). (DOCX 23 kb) [file 40900_2017_60_MOESM1_ESM.docx]

**ADDITIONAL FILE 1: Medline Search strategy (Ovid)**

1. ((public or service or clients$ or user or users or patient or patients or consumer or consumers or carer or carers) adj4 (involve$ or engag$ or participat$ or action)).af.

2. ((stakeholder$ or advisor$ or reference$ or expert$ or consultation or steering) adj2 (group$ or panel)).af.

3. ((charit$ or non-profit or third) adj2 (organi?ation or group or sector)).af.

4. or/1-3

5. meta-analysis.pt.

6. meta-analysis/ or systematic review/ or meta-analysis as topic/ or "meta analysis (topic)"/ or "systematic review (topic)"/ or exp technology assessment, biomedical/

7. ((systematic$ adj3 (review$ or overview$)) or (methodologic$ adj3 (review$ or overview$))).ti,ab.

8. ((quantitative adj3 (review$ or overview$ or synthes$)) or (research adj3 (integrati$ or overview$))).ti,ab.

9. ((integrative adj3 (review$ or overview$)) or (collaborative adj3 (review$ or overview$)) or (pool$ adj3 analy$)).ti,ab.

10. (data synthes$ or data extraction$ or data abstraction$).ti,ab.

11. (handsearch$ or hand search$).ti,ab.

12. (mantel haenszel or peto or der simonian or dersimonian or fixed effect$ or latin square$).ti,ab.

13. (met analy$ or metanaly$ or technology assessment$ or HTA or HTAs or technology overview$ or technology appraisal$).ti,ab.

14. (meta regression$ or metaregression$).ti,ab.

15. (meta-analy$ or metaanaly$ or systematic review$ or biomedical technology assessment$ or bio-medical technology assessment$).mp,hw.

16. (medline or cochrane or pubmed or medlars or embase or cinahl).ti,ab,hw.

17. (cochrane or (health adj2 technology assessment) or evidence report).jw.

18. (meta-analysis or systematic review).mp.

19. (comparative adj3 (efficacy or effectiveness)).ti,ab.

20. (outcomes research or relative effectiveness).ti,ab.

21. ((indirect or indirect treatment or mixed-treatment) adj comparison$).ti,ab.

22. (meta-ethnograph$ or metaethnograph$ or meta ethnograph or meta-study or metastudy or meta study).ti,ab.

23. ((qualitative adj3 (review$ or overview$ or synthes$)) or (research adj3 (integrati$ or overview$))).ti,ab.

24. (evidence or realist adj3 (review$ or overview$ or synthes$)).ti,ab.

25. or/5-24

26. 4 and 25

27. limit 27 to yr="2014 -Current"
